# A Novel Estimation of Unobserved Pig Growth Traits for the Purposes of Precision Feeding Methods

**DOI:** 10.3389/fvets.2021.689206

**Published:** 2021-07-29

**Authors:** Maciej M. Misiura, Joao A. N. Filipe, Ilias Kyriazakis

**Affiliations:** ^1^Newcastle University, Newcastle upon Tyne, United Kingdom; ^2^Biomathematics & Statistics Scotland, Rowett Institute of Nutrition and Health, University of Aberdeen, Aberdeen, United Kingdom; ^3^Biological Sciences Building, Queen's University Belfast, Belfast, United Kingdom

**Keywords:** Bayesian inference, body composifion, individual traits, nitrogen excretion, phosphorus excretion, precision feeding, pigs

## Abstract

Recent technological advances make it possible to deliver feeding strategies that can be tailored to the needs of individual pigs in order to optimise the allocation of nutrient resources and contribute toward reducing excess nutrient excretion. However, these efforts are currently hampered by the challenges associated with: (1) estimation of unobserved traits from the available data on bodyweight and feed consumption; and (2) characterisation of the distributions and correlations of these unobserved traits to generate accurate estimates of individual level variation among pigs. Here, alternative quantitative approaches to these challenges, based on the principles of inverse modelling and separately inferring individual level distributions within a Bayesian context were developed and incorporated in a proposed precision feeding modelling framework. The objectives were to: (i) determine the average and distribution of individual traits characterising growth potential and body composition in an empirical population of growing-finishing barrows and gilts; (ii) simulate the growth and excretion of nitrogen and phosphorus of the average pig offered either a commercial two-phase feeding plan, or a precision feeding plan with daily adjustments; and (iii) simulate the growth and excretion of nitrogen and phosphorus across the pig population under two scenarios: a two-phase feeding plan formulated to meet the nutrient requirements of the average pig or a precision feeding plan with daily adjustments for each and every animal in the population. The distributions of mature bodyweight and ratio of lipid to protein weights at maturity had median (IQR) values of 203 (47.8) kg and 2.23 (0.814) kg/kg, respectively; these estimates were obtained without any prior assumptions concerning correlations between the traits. Overall, it was found that a proposed precision feeding strategy could result in considerable reductions in excretion of nitrogen and phosphorus (average pig: 8.07 and 9.17% reduction, respectively; heterogenous pig population: 22.5 and 22.9% reduction, respectively) during the growing-finishing period from 35 to 120 kg bodyweight. This precision feeding modelling framework is anticipated to be a starting point toward more accurate estimation of individual level nutrient requirements, with the general aim of improving the economic and environmental sustainability of future pig production systems.

## Introduction

To address economic and environmental concerns about standard feeding practises in commercial pig production (1–3), precision feeding strategies have been suggested as a way forward (4–6). Precision feeding strategies aim to accurately match nutrient supply to the demand of animals by formulating feeds that account for the dynamic changes in nutrient requirements, preferably at the individual level (7, 8). This is in contrast to standard feeding practises, which typically neglect variation in nutrient requirements among individuals, as they involve formulating feeds that satisfy the estimated nutrient requirements of a nominal average pig in a population, at a given static reference point specified by bodyweight (*BW*) or age (9). Initial evaluations of precision feeding strategies against standard population level feeding regimes in growing-finishing pigs have been encouraging based on reports of considerable reduction in excretion of nitrogen (N) and phosphorus (P), without any apparent loss in growth performance (10–12).

A successful implementation of precision feeding requires the development of methods for estimating the nutrient requirements of individual pigs, which in turn requires estimating their growth potential and body composition. There are notable issues associated with this challenge, which concern: (1) estimation of unobserved traits from data; and (2) characterisation of the distributions and correlations of these unobserved traits. Regarding the first issue, while body composition is a major determinant of nutrient requirements, real-time data on e.g., protein or lipid retention are either rare or unavailable due to technological and logistical limitations (13–15), and consist of tissue scan proxies with limited correlation to body amounts (16). Consequently, these traits are often estimated from data on *BW* and feed consumption by making assumptions whose validity could be restrictive. This limits the ability to formulate optimal feeding rations. For example, a typical approach to obtain information on lean tissue growth and requirements for precision feeding of growing pigs assumes an isometric relationship relating protein retention and *BW* gain, and that the isometric parameters are the same across pigs (5). However, this approach neglects individual variation in protein growth among animals as well as the non-proportionality between these variables during growth (17, 18). Alternative models, including a polynomial regression relating body protein weight to *BW* have been recently developed (19), but their validity is still largely unascertained.

Regarding the issue of the distributions and correlations of the unobserved traits of individuals, a typical approach relies on an explicit specification of their multivariate distribution (20–26). Within this setting, it is necessary to either assume or estimate multiple mean and variance-covariance parameters from data, which carries uncertainty (27) and can be challenging in practise (9). To avoid these challenges, a potential alternative approach to model trait variation, based on separately inferring individual level distributions within a Bayesian framework, has been recently suggested by Filipe and Kyriazakis (27). This framework is yet to be comprehensively tested in the context of the estimation of traits that are typically required for precision feeding purposes.

The aims of this chapter were to develop alternative data-driven approaches to estimate uncertain traits in individual pigs and incorporate this information in a proposed precision feeding modelling framework. This modelling framework was applied to evaluate feeding strategies in their effectiveness to minimise excess excretion of N and P when compared to standard feeding practises. These evaluations were conducted by considering the average of the individual responses in a population and the response of the average pig in the population, to gain a better insight into possible impacts of phenotypic heterogeneity on nutrient excretion. The specific objectives were to: (1) determine the empirical average and distribution of individual traits characterising growth potential and body composition in a pig population; (2) simulate the growth and excretion of N and P of the average pig offered either a commercial two-phase feeding plan, or a precision feeding plan with daily adjustments; and (3) simulate the growth and excretion of N and P across the pig population under two scenarios: a two-phase feeding plan formulated to meet the nutrient requirements of the average pig or a precision feeding plan with daily adjustments for each and every animal in the population.

## Materials and Methods

There was no requirement for ethical approval, since the data used originated from a previous experiment, which was granted ethical approval on behalf of the original trial investigators.

### Data

Empirical sequential data on individual daily feed intake *DFI*_*t*_ (kg/d) and *BW*_*t*_ (kg), at ages *t* (d), of 32 barrows and gilts [(Large White × Landrace) × Pietrain] were obtained from a trial conducted by the INRAE at the UE3P unit (Pig Physiology and Phenotyping Experimental Facility, https://doi.org/10.15454/1.5573932732039927E12), Saint Gilles, France. Pigs were kept in near-commercial conditions (*ad-libitum* access to water and feeds, group housing, ambient room temperature of 20–24°C) for a period of 81 d from an initial mean *BW* of 35.2 (SD: 4.70) kg until a final mean *BW* of 118 (SD: 8.87) kg. The pigs were given access to two feeds in succession formulated to meet or exceed the expected population level average nutritional requirements. The change in feeds occurred when animals reached ~65.0 kg.

### Approach to Estimate Individual Level Variation in Growth Potential and Body Composition

#### Model Description

The Gompertz growth model (28), comprehensively reviewed by Filipe et al. (29), was used to describe the evolution of *BW*_*t*_ of each individual pig over time:

(1.1) $$\begin{array}{l} BW_{t}=BW_{m}\times \exp\left(-\ln\;\left(\frac{BW_{m}}{BW_{in}}\right)\times \exp\left(-\frac{t-t_{0}}{B}\right)\right)\left(\text{kg}\right) \end{array}$$

where *t* and *t*_0_ were the current and initial times (d), *BW*_*in*_ (kg) was the observed initial bodyweight at the start of the data collection period, and *BW*_*m*_ (kg) and *B* (d) were unknown parameters (traits) estimated for each pig. The unknown model traits correspond to the weight at maturity and the inverse of the growth rate controlling how fast the weight at maturity is reached.

After accounting for gut fill to derive the empty *BW*, *eBW*_*t*_ (30), this *eBW*_*t*_ was expressed as a sum of the four main body chemical components (31): protein [N^*^ = 6.25 × × N, where N is nitrogen (kg)], lipid (*L*) (kg), water (*W*) (kg) and ash (Ash) (kg):

(1.2) $$\begin{array}{l} eBW_{t}=\alpha \times BW_{t}=N_{t}^{*}+L_{t}+W_{t}+Ash_{t}\left(\text{kg}\right) \end{array}$$

where α was assumed to be a constant proportion over the growth period under consideration, equal to 95% of *BW*_*t*_ (32, 33) and to be the same across animals.

The growth of these four body chemical components was represented by the following allometric relationships (29, 34–36):

(1.3) $$\begin{array}{l} N_{t}^{*}=N_{m}^{*}\times {\left(\frac{BW_{t}}{BW_{m}}\right)}^{\frac{\log\left(N_{m}^{*}/N_{in}^{*}\right)}{\log\left(BW_{m}/BW_{in}\right)}}\left(\text{kg}\right) \end{array}$$

(1.4) $$\begin{array}{l} L_{t}=L_{m}\times {\left(\frac{BW_{t}}{BW_{m}}\right)}^{\frac{\log\left(L_{m}/L_{in}\right)}{\log\left(BW_{m}/BW_{in}\right)}}\left(\text{kg}\right) \end{array}$$

(1.5) $$\begin{array}{l} W_{t}=3.04\times {\left(\frac{N_{t}^{*}}{N_{m}^{*}}\right)}^{0.855}\left(\text{kg}\right) \end{array}$$

(1.6) $$\begin{array}{l} Ash_{t}=0.190\times \left(\frac{N_{t}^{*}}{N_{m}^{*}}\right)\left(\text{kg}\right) \end{array}$$

where $N_{m}^{*}$ and *L*_*m*_ are mature weights, and $N_{in}^{*}$ and *L*_*in*_ are initial weights of protein and lipid, respectively; these traits were unknown in advance and had to be estimated from individual data from each pig in the population.

#### Fitting to the Data

To estimate the traits characterising each individual pig in the population, Equations (1.1–1.6) describing the dynamic evolution of *BW*_*t*_,$N_{t}^{*},\;L_{t},\;W_{t},\;$and *Ash*_*t*_ were fitted to the data of each individual pig one at a time.

To account for the uncertainty and correlations between individual trait estimates, a Bayesian inference approach was utilised, which outputs estimated distributions rather than point estimates of the traits (37). Samples of trait estimates were obtained using the Markov Chain Monte Carlo (MCMC) methods (38) and more specifically, the Metropolis-Hastings algorithm (39). The posterior inferences on the traits were based on samples generated using the MCMC engine *rjags* (40). Prior distributions for the traits are given in the Supplementary Material, together with a justification for their choice. Four independent MCMC chains, each containing 100,000 samples and initialised with different random starting parameter values, were generated, from which the first ten percent samples were discarded as burn-in (41, 42). The posterior inferences were carried out on the remaining 90,000 samples from each chain; no thinning was applied (43). Four MCMC chains, rather than one, were used as a way of assessing differences among the sampled trait distributions and thus, was a first convergence diagnostic (44). The convergence of each sample chain was also assessed by investigating trace plots (after burn-in) for each trait and by calculating the potential scale reduction factor, $\hat{R}$ (45, 46). Values of $\hat{R}$. > 1.01 were considered to indicate poor convergence (47). The posterior distribution of sampled traits used for inference comprised every chain that converged; for example, when the four chains converged, it comprised *N*_*s*_ = 4 × 90,000 = 360,000 sampled trait values.

#### Data-Based Estimation of the Average Pig in the Population

The average pig in the population was estimated by minimising the following metric across the pigs in the population:

(1.7) $$\begin{array}{l} D_{i}=\overset{4}{\underset{j=1}{\sum }}\left|\frac{{Ŷ}_{ij}-{Ȳ}_{j}}{{Ȳ}_{j}}\right| \end{array}$$

Where Ŷ_*ij*_ are obtained estimates of the traits $BW_{m},B,N_{m}^{*}$ and *L*_*m*_ for pig *i* (*i* = 1, …, 32) in the population, and Ŷ_*j*_ are the median values of these trait estimates calculated across the population. The pig whose set of estimates Ŷ_*j*_ had the lowest value of *D* was chosen to characterise the average pig as its traits were regarded as central in the population. This specific approach to multidimensional estimation of the average pig was chosen because it preserves the individual level correlations between traits which were estimated jointly for each animal in the population (27).

### Estimation of Nutrient Requirements

Daily requirements for N^*^, P and energy of the estimated average pig and of each pig in the pig population (whose individual traits were estimated) were expressed as a sum of requirements for maintenance and growth using the equations in Table 1; inputs to these equations were the data-driven trait estimates that are the parameters of Equations (1.1–1.6).

**Table 1:** Equations to estimate individual daily requirements for maintenance and growth in terms of effective energy (*E*), digestible protein (N^*^) and digestible phosphorus (P).

**Quantity**	**Abbreviation**	**Equation**	**Unit**	**Efficiency value**	**Source**
Energy	*E*_*maint*_(*t*)	$\frac{1}{e_{E_{m}}}\times \left(1.63\times \frac{{\text{N}}^{*}(t)}{N_{m}^{*}{\;}^{0.27}}\right)$	(MJ/d)	*e*_*E*_*m*__ = 1.00	(31)
Protein	$N_{maint}^{{*}^{{\;}^{\prime }}}\left(t\right)$	$\frac{1}{e_{N^{{*}^{{\;}^{\prime }}}}{\;}_{m}}\times \left(0.004\times \frac{{\text{N}}^{*}(t)}{N_{m}^{*}{\;}^{0.27}}\right)$	(kg/d)	${e_{N^{{*}^{{\;}^{\prime }}}}}_{m}=1.00$	(30)
Phosphorus	${P^{{\;}^{\prime }}}_{maint}\left(t\right)$	$\frac{1}{e_{P_{m}^{{\;}^{\prime }}}}\times \left(0.0001293\times \frac{{\text{N}}^{*}(t)}{N_{m}^{*}{\;}^{0.27}}\right)$	(kg/d)	$e_{P_{m}^{{\;}^{\prime }}}=1.00$	(30)
Energy	*E*_*growth*_(*t*)	$e_{E_{g_{N^{*}{\;}^{{\;}^{\prime }}}}}\times N_{max}^{{*}^{{\;}^{\prime }}}(t)+e_{E_{g_{L^{{\;}^{\prime }}}}}\times L\prime_{max}(t)$	(g/kg)	$e_{E_{g_{{N^{*}}^{{\;}^{\prime }}}}}=50.0;e_{E_{g_{L^{{\;}^{\prime }}}}}=56.0$	(30)
Protein	$N_{growth_{max}}^{{*}^{{\;}^{\prime }}}\left(t\right)$	$\frac{1}{e_{N_{g}^{{*}^{{\;}^{\prime }}}}}\times N_{max}^{{*}^{{\;}^{\prime }}}\left(t\right)$	(kg/d)	$e_{N_{g}^{{*}^{{\;}^{\prime }}}}=0.763$	(48)
Phosphorus	$P_{growth_{max}}^{{\;}^{\prime }}\left(t\right)$	$\frac{1}{e_{P_{g}^{{\;}^{\prime }}}}\times P_{max}^{{\;}^{\prime }}\left(t\right)$	(kg/d)	$e_{P_{g}^{{\;}^{\prime }}}=0.940$	(49)

${\text{N}}_{\text{max}}^{{\text{*}}^{{\;}^{\prime }\;}}\left(\text{t}\right)=\;\frac{1}{\text{B}}\times {\text{N}}_{\text{t}}^{\text{*}}\times log\left(\frac{{\text{N}}_{\text{m}}^{\text{*}}}{{\text{N}}_{\text{t}}^{\text{*}}}\right)$ is the daily maximum retention of N^*^ at time t; ${\text{P}}_{\text{max}}^{{\;}^{\prime }\;}\left(\text{t}\right)=\;0.0337\times \;\frac{1}{\text{B}}\times {\text{N}}_{\text{t}}^{\text{*}}\times log\left(\frac{{\text{N}}_{\text{m}}^{\text{*}}}{{\text{N}}_{\text{t}}^{\text{*}}}\right)\;$is the daily maximum retention of P at time t; ${{\text{L}}^{{\;}^{\prime }\;}}_{\text{max}}\left(\text{t}\right)=\;\frac{1}{\text{B}}\times {\text{L}}_{\text{t}}\times log\left(\frac{{\text{L}}_{\text{m}}}{{\text{L}}_{\text{t}}}\right)$. is the daily desired (normal) retention of lipid at time t.

Maintenance requirements for N^*^, P and energy at *t* were related to the estimated $N_{t}^{*}$ and $N_{m}^{*}$, rather than *BW*_*t*_ and *BW*_*m*_, to account for any potential variation in these requirements due to differences in body composition among animals (30). It was assumed that there were no inefficiencies in utilising these nutrients for maintenance purposes (48, 49).

Growth requirements for N^*^, P and energy at *t* were related to the maximum daily retention of N^*^ (kg/d) and P (kg/d) and to the desired (normal) retention of *L* (kg/d), which were estimated as:

(1.8) $$\begin{array}{l} N_{max}^{{*}^{\prime }}\left(t\right)=\;\frac{1}{B}\times N_{t}^{*}\times \log\left(\frac{N_{m}^{*}}{N_{t}^{*}}\right) \end{array}$$

(1.9) $$\begin{array}{l} P_{max}^{{\;}^{\prime }}\left(t\right)=\;0.0337\times \;\frac{1}{B}\times N_{t}^{*}\times \log\left(\frac{N_{m}^{*}}{N_{t}^{*}}\right) \end{array}$$

(1.10) $$\begin{array}{l} L\prime_{max}(t)=\;\frac{1}{B}\times L_{t}\times \log\left(\frac{L_{m}}{L_{t}}\right) \end{array}$$

To calculate growth requirements, equations (1.8–1.10) were multiplied by coefficients that account for the metabolic inefficiencies in the utilisation of nutrients for retention processes (50–52) and thus, to derive requirements expressed on digestible N^*^ (30) (kg/d), digestible P (kg/d) (49) and effective energy basis (MJ/d), which is the difference between digestible energy and losses associated with feed consumption (53).

### Simulated Feeding Scenarios

Four feeding scenarios were considered to quantify the effects on N and P excretion of the within- and between- animal variation in growth potential and body composition. The first two scenarios were designed to predict differences in growth performance, and N and P excretion of the average pig offered either a “static” feeding strategy that targeted its nutrient requirements at pre-specified reference points, or a precision feeding strategy that adapted to the dynamic evolution of the performance of this animal. These two scenarios are equivalent to investigating responses of the homogeneous pig population. The remaining two scenarios were designed to quantify differences in growth performance, and in N and P excretion across the heterogenous pig population offered either a “static” feeding strategy that targeted nutrient requirements of the average animal or a precision feeding strategy that adapted to real-time performance of each individual pig within the population.

#### Scenario 1: Two-Phase Feeding Strategy for the Average Pig

The first scenario (S1) simulated the growth of the average pig from 35.0 to 120 kg when given *ad-libitum* access to two feeds (Feed 1 and Feed 2), offered in succession with a switch from Feed 1 to Feed 2 at ~65.0 kg. The nutritional composition of Feed 1 and Feed 2, in terms of crude N^*^ (g/kg), digestible N^*^ (g/kg), total P (g/kg), digestible P (g/kg), and effective energy (MJ/kg) were inputs into the growth model. The following contents were calculated by dividing the estimated nutrient requirements for maintenance and growth of the average pig at reference points *t*_*i*_ by the median *DFI*_*t*_ at the same point from the collected data across the thirty-two pigs (section Data):

(1.11) $$\begin{array}{l} X_{t_{i}}=\frac{X_{maint}\left(t_{i}\right)+X_{growth}\left(t_{i}\right)}{DFI_{t_{i}}} \end{array}$$

where *X*= [digestible N^*^, digestible P, effective energy] and *t*_*i*_ = (1,2) are the reference points where *BW*_*t*_1__ = 50.0 kg (Feed 1) or *BW*_*t*_2__ = 92.5 kg (Feed 2), which are based on Symeou et al. (54). Crude N^*^ contents were calculated according to Wellock et al. (55) by dividing digestible N^*^ contents in each feed by the product of the digestibility coefficient, 0.800, and the biological value [a common measure of N^*^ quality in the feed (56)], 0.750, reflective of typical commercial feeds (57). Total P contents were calculated by dividing digestible P contents in each feed by the digestibility coefficient, equal to 0.500 (58) to derive total *P* values consistent with the typical commercial feeds (26). As nutrient requirements for the average pig were conditional on the estimates under section Estimation of nutrient requirements, estimated nutritional composition of Feed 1 and Feed 2 is given in section Estimation of nutrient requirements and feed composition.

#### Scenario 2: Precision Feeding Strategy for the Average Pig

The second scenario (S2) simulated the growth of the average pig given *ad-libitum* access to feeds adjusted daily for a time period (d) equal to the length of S1. Daily adjustments to the nutritional composition of the feeds, in terms of digestible N^*^, digestible P and effective energy were calculated as the ratio of the estimated daily nutrient requirements for maintenance and growth of the average pig to the estimated target *DFI*_*t*_ of this pig. The target *DFI*_*t*_ was estimated using:

(1.12) $$\begin{array}{l} DFI_{t}=\theta_{1}\left(CG_{t}^{\theta_{2}}-CG_{t-1}^{\theta_{2}}\right), \end{array}$$

where *CG* is the cumulative gain, θ_1_ and θ_2_ are parameters estimated from the animal's past *BW* and feed consumption. Crude N^*^ feed contents were calculated according to Wellock et al. (55) by dividing digestible N^*^ contents in each feed by the product of the digestibility coefficient, equal to 0.800 and the biological value, equal to 0.750. Total P contents in Feed 1 and Feed 2 were calculated by dividing digestible P contents in each feed by the digestibility coefficient, equal to 0.500 to derive total *P* values. It is assumed that the usual practise of blending high-nutrient and low-nutrient basal feeds would not alter nutrient composition of these feeds. However, blend feeding was not explicitly considered in this study.

#### Scenario 3: Two-Phase Feeding Strategy for the Heterogenous Pig Population

The third scenario (S3) extended S1 to the heterogenous pig population by simulating the growth of each pig in the population for a time period from the population average *BW* of 35.0–120 kg. Each pig in the population was given *ad-libitum* access to Feed 1 and Feed 2, with a change in feeds when the population average *BW* reached 65.0 kg.

#### Scenario 4: Precision Feeding Strategy for the Heterogenous Pig Population

The fourth scenario (S4) extended S2 in the context of the heterogenous pig population. This scenario simulated the growth of each pig in the population offered *ad-libitum* access to the individualised precision feeding plan, adjusted daily to adapt to real-time performance of each pig, for a time period equal to the length of S3. For each pig, daily adjustments to the nutritional composition of the feeds were calculated using the approach described in section Scenario 2: precision feeding strategy for the average pig but accounting for the individualised daily nutrient requirements for maintenance and growth, and target *DFI*_*t*_.

### Estimation of Growth and Nutrient Excretion

In S1 and S3, which described the commercial two-phase feeding strategy, Feed 1 and Feed 2 were assumed to result in periods of nutrient under-supplementation or over-supplementation for a number of pigs (54). When undersupplied with nutrients, pigs were assumed to consume excess amounts of feeds when either N^*^or energy was the most deficient (20, 59), as an attempt to eat for the first limiting feed resource in the feed, but not when P was the most deficient (49, 60). In the cases of P deficiencies, feed intake was assumed to be controlled only by the energy needed to support the potential growth. In S2 and S4, which described the precision feeding strategy, the individualised feeds were assumed to provide the precise quantities of nutrients to support maintenance and growth requirements of each pig.

Daily feed consumption, *DFI*_*t*_ was predicted using the following equation:

(1.13) $$\begin{array}{l} DFI_{t}=\{\begin{array}{c} \frac{E_{maint}\left(t\right)+E_{growth}\left(t\right)}{E_{feed}},\text{ energy or P limiting}\\ \frac{N_{maint}^{{*}^{\prime }}\left(t\right)+N_{growth_{max}}^{{*}^{\prime }}\left(t\right)}{N_{feed}^{*}},\text{ protein limiting} \end{array} \end{array}$$

where terms in the numerator of this equation are given in Table 1, and *E*_*feed*_ and $N_{feed}^{*}$ are effective energy feed content (MJ/kg) and digestible N^*^ (g/kg), respectively. There were no additional constraints (such as bulkiness of the feed) imposed on the actual feed consumption and pigs were assumed to be kept in a thermoneutral housing environment (61). The predicted *DFI*_*t*_ was utilised to inform the actual growth, which could differ from the potential growth. The actual retention of protein (N^*^′(*t*)) and retention of P (*P*′(*t*)) were determined by the actual *DFI*_*t*_ function used but these quantities were assumed to not exceed $N_{max}^{{*}^{{\;}^{\prime }}}\left(t\right)$ or $P_{max}^{{\;}^{\prime }}\left(t\right)$, respectively. Any excess N^*^ consumed was assumed to be deaminated and excreted as urea (53); any excess energy was assumed to be retained as excess *L* (62). The actual *L* retention was calculated as follows:

(1.14) $$\begin{array}{l} L^{{\;}^{\prime }}\left(t\right)=\frac{DFI_{t}\times E_{feed}-E_{maint}\left(t\right)-E_{N}\times N^{{*}^{\prime }}\left(t\right)}{E_{L}} \end{array}$$

where *E*_*N*_ and *E*_*L*_ are the energy used (and expressed in effective energy scale) per kg of N^*^ and *L* retained, respectively. The retention of *A*sh and *W* were related to N^*^′ and implemented as in Wellock et al. (30) and Symeou et al. (49).

Daily excretion of N (*N*_*out*_(*t*)) (kg/d) and P (*P*_*out*_(*t*)) (kg/d) were calculated as follows:

(1.15) $$\begin{array}{l} N_{out}\left(t\right)=\frac{\left(\left(DFI_{t}\times \frac{\text{crude }N^{*}}{1000}\right)-N_{maint}^{{*}^{\prime }}\left(t\right)-N^{{*}^{\prime }}\left(t\right)\right)}{6.25} \end{array}$$

(1.16) $$\begin{array}{l} P_{out}\left(t\right)=\left(DFI_{t}-\frac{\text{total }P}{1000}\right)-{P^{{\;}^{\prime }}}_{maint}\left(t\right)-P^{{\;}^{\prime }}\left(t\right) \end{array}$$

where crude N^*^ and total P denote the feed levels of these quantities per kg of feed.

### Simulated Outputs

The following outputs were generated to assess growth performance and nutrient excretion of either the average pig (S1 and S2) or of the heterogenous pig population (S3 and S4): (1) average daily feed intake (ADFI; kg/d/pig); (2) average daily gain (ADG; kg/d/pig); (3) feed conversion ratio (FCR; kg/kg/pig); (4) average daily N^*^ retention (kg/d/pig); (5) average daily *L* retention (kg/d/pig); (6) final N^*^ weight at end of each simulation (kg/pig); (7) final *L* weight at end of each simulation (kg/pig); (8) cumulative N and P balances [intake, retention, excretion (kg/pig)]. For S1 and S2, the outputs were expressed in terms of mean values; for S3 and S4 the outputs were expressed in terms of mean (SD) values.

## Results

### Data-Based Estimation of the Average Pig and the Heterogenous Pig Population

**Figure 1:**
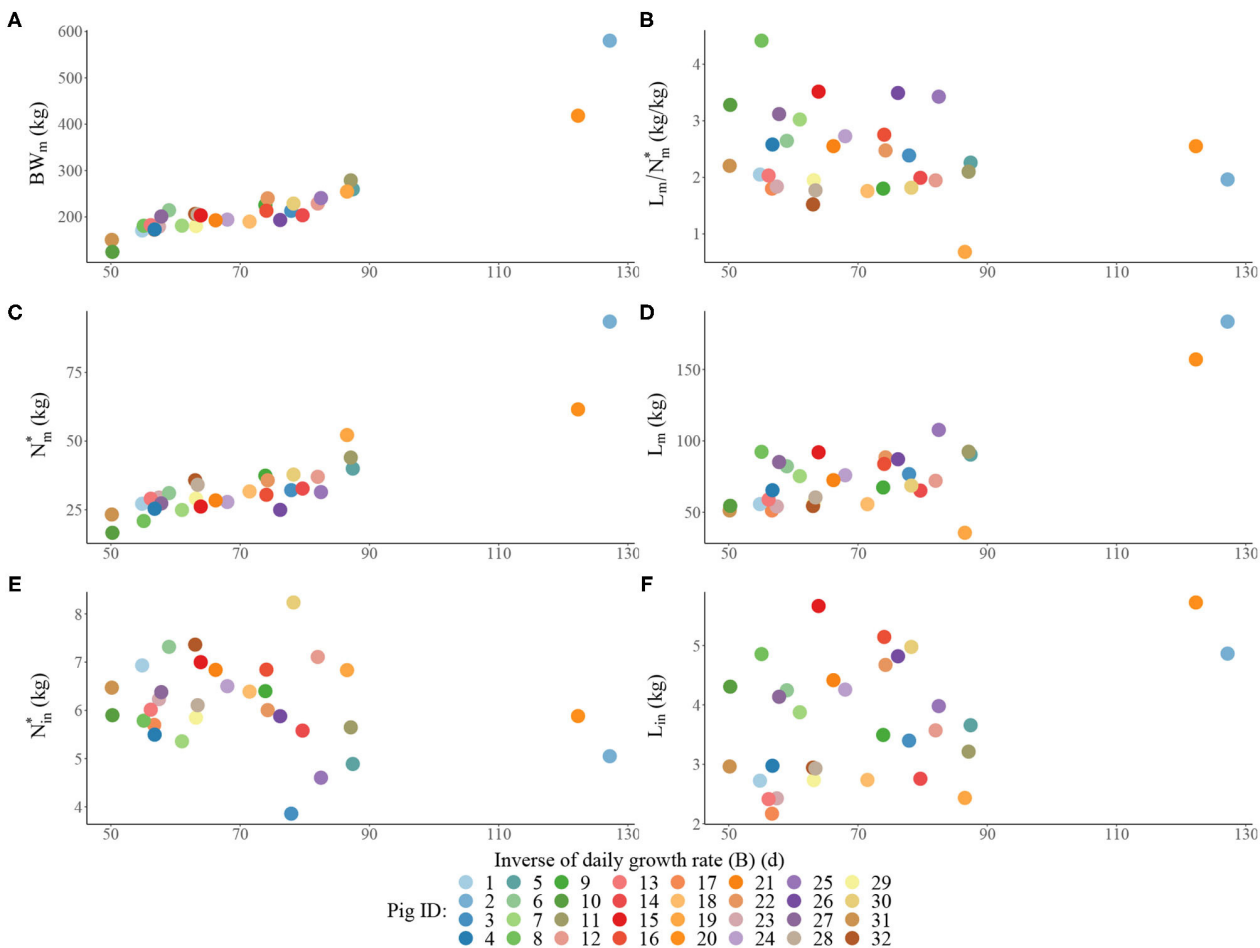
Scatterplots of the estimated traits for each individual pig in the population: **(A)** mature bodyweight (*BW*_*m*_) vs. inverse of daily growth rate *B*B; **(B)** ratio of lipid to protein weights at maturity $\left(L_{m}/N_{m}^{*}\right)$ vs. *B*; **(C)** mature protein weight ($N_{m}^{*}$) vs. *B*; **(D)** mature lipid weight (*L*_*m*_) vs. *B*; **(E)** initial protein weight ($N_{in}^{*}$) vs. *B*; **(F)** initial lipid weight (*L*_*in*_) vs. *B*.

Estimated traits for each pig in the heterogenous population are visualised in Figure 1 and are summarised by the descriptive statistics calculated across the individuals in Table 2. Within the population: (1) *BW*_*m*_ ranged from 124 to 580 kg; (2) B ranged from 50.1 to 127 d; (3) $L_{m}/N_{m}^{*}$ ranged from 0.683 to 4.41 (kg/kg); (4) $N_{m}^{*}$ ranged from 16.6 to 93.5 kg; (5) *L*_*m*_ ranged from 35.7 to 184 kg; (6) $N_{in}^{*}$ ranged from 3.86 to 8.24 kg; (7) *L*_*in*_ ranged from 2.17 to 5.72 kg. There were three pigs that were notably different from the remaining animals in the population, namely: (i) two pigs were notably larger at maturity than the rest, with the estimated *BW*_*m*_ exceeding 400 kg (Figure 1A); (ii) one pig was notably leaner than the rest, with the estimated $L_{m}/N_{m}^{*}$ below one (Figure 1B). Despite these differences, these three potential outlier pigs were kept in further analyses as their inclusion or exclusion did not influence the overall comparisons of different feeding strategies (see Supplementary Material for results produced in the context of pig population which excluded the three aforementioned pigs). Estimated traits of the average pig in the population were: *BW*_*m*_ = 205 kg; *B* = 65.0 days;$\;L_{m}/N_{m}^{*}=2.31\;$(kg/kg); $N_{m}^{*}=31.0\;$ kg; *L*_*m*_ = 71.6 kg; $N_{in}^{*}=7.33$ kg; *L*_*in*_ = 3.34 kg.

**Table 2:** Summary statistics of the estimated traits across the thirty-two pigs in the population.

**Trait**	**Min**	**Median**	**IQR**	**Mean**	**SD**	**Mode**	**Max**
*BW*_*m*_ (kg)	124	203	47.8	221	82.2	226	580
B (days)	50.1	67.1	20.9	71.4	17.9	73.9	127
$L_{m}/N_{m}^{*}$ (kg/kg)	0.683	2.23	0.814	2.39	0.737	1.80	4.41
$N_{m}^{*}$ (kg)	16.6	30.8	8.78	34.0	13.9	37.4	93.5
*L*_*m*_ (kg)	35.7	72.3	31.0	77.2	29.5	67.4	184
$N_{in}^{*}$ (kg)	3.86	6.06	1.115	6.14	0.880	6.40	8.24
*L*_*in*_ (kg)	2.17	3.61	1.59	3.73	1.01	3.49	5.72

BW_m_, mature bodyweight; B, inverse of daily growth rate; ${\text{L}}_{\text{m}}/{\text{N}}_{\text{m}}^{\text{*}}$, ratio of lipid to protein weights at maturity; ${\text{N}}_{\text{m}}^{\text{*}}$, mature protein weight; L_m_, mature lipid weight; ${\text{N}}_{\text{in}}^{\text{*}}$, initial protein weight; L_in_, initial lipid weight.

### Estimation of Nutrient Requirements and Feed Composition

For the two-phase feeding strategies under consideration (S1 and S3), the kg of Feed 1 was estimated to contain 181 g of crude N^*^, 109 g of digestible N^*^, 6.01 g of total P, 3.01 g of digestible P and 11.8 MJ of effective energy, in order to meet precisely the requirements of this pig at the mid-point of the period under consideration. Subsequently, the kg of Feed 2 was estimated to contain 122 g of crude N^*^, 72.9 g of digestible N^*^, 4.06 g of total P, 2.03 g of digestible P and 11.8 MJ of effective energy.

**Figure 2:**
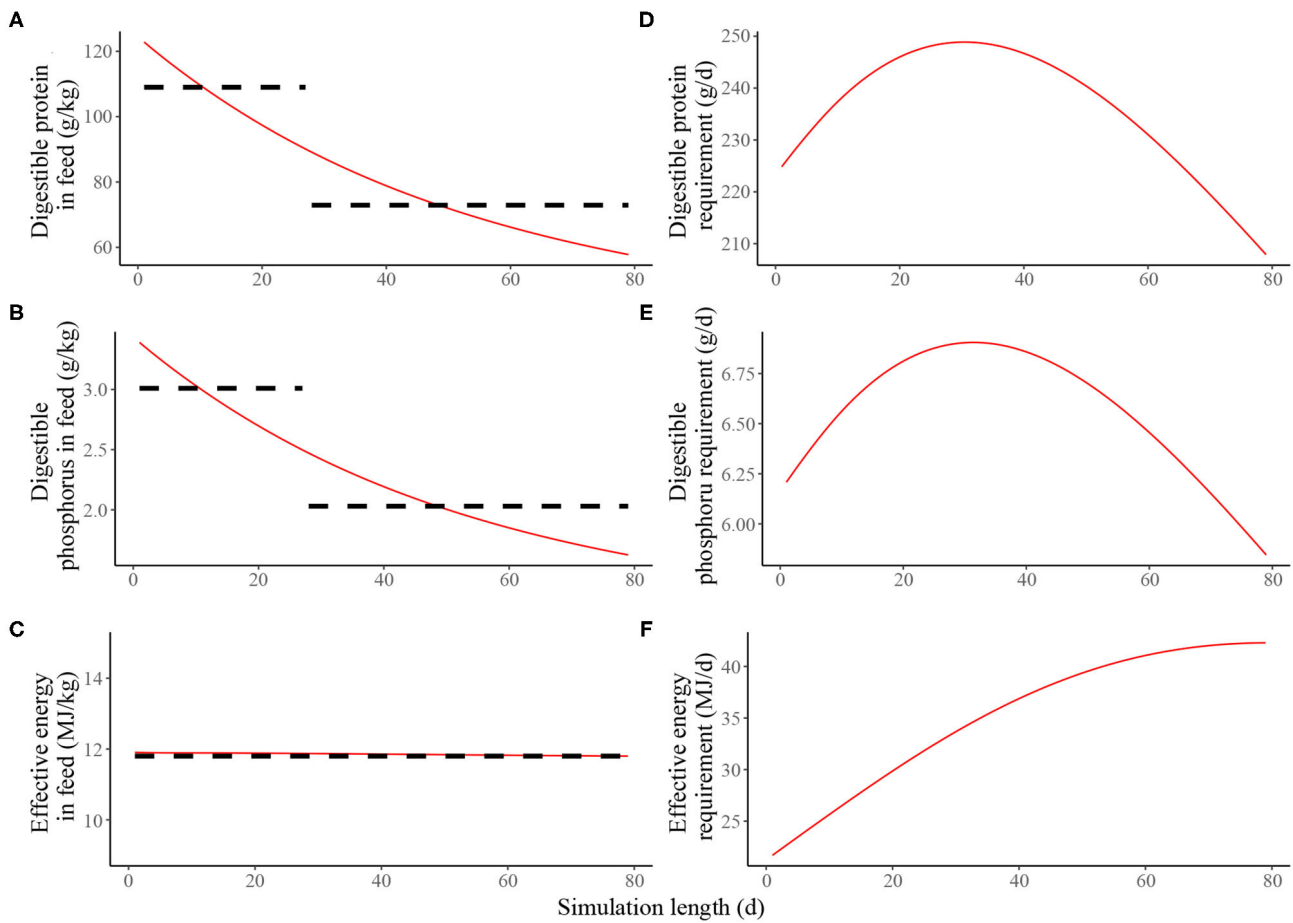
Estimated nutritional composition of the feeds in either two-phase feeding strategy (dashed black line, Scenario 1) or precision feeding strategy (solid red line; Scenario 2) offered to the average pig in terms of: **(A)** digestible protein (g/kg); **(B)** digestible phosphorus (g/kg); **(C)** effective energy (MJ/kg); estimated daily nutrient requirement for maintenance and growth of the average pig in terms of: **(D)** digestible protein (g/d); **(E)** digestible phosphorus (g/d); **(F)** effective energy (MJ/d). For a detailed description of the simulated scenarios, see section Simulated Feeding Scenarios.

Estimated nutritional composition of the feeds (in terms of digestible N^*^, digestible P and effective energy) in S1 and S2, together with the accompanying estimated daily nutrient requirements for maintenance and growth of the average pig is given in Figure 2. In the context of the average pig, the precision feeding strategy (S2) resulted in gradual decreases in digestible N^*^ and digestible P feed contents over time; the effective energy content of the feeds remained largely unchanged over time. On the first day of S2, the kg of feed was estimated to contain 205 g of crude N^*^, 123 g of digestible N^*^, 6.78 g of total P, 3.39 g of digestible P and 11.9 MJ of effective energy. On the last day, the kg of feed was estimated to contain 96.3 g of crude N^*^, 57.8 g of digestible N^*^, 3.25 g of total P, 1.62 g of digestible P and 11.8 MJ of effective energy.

**Figure 3:**
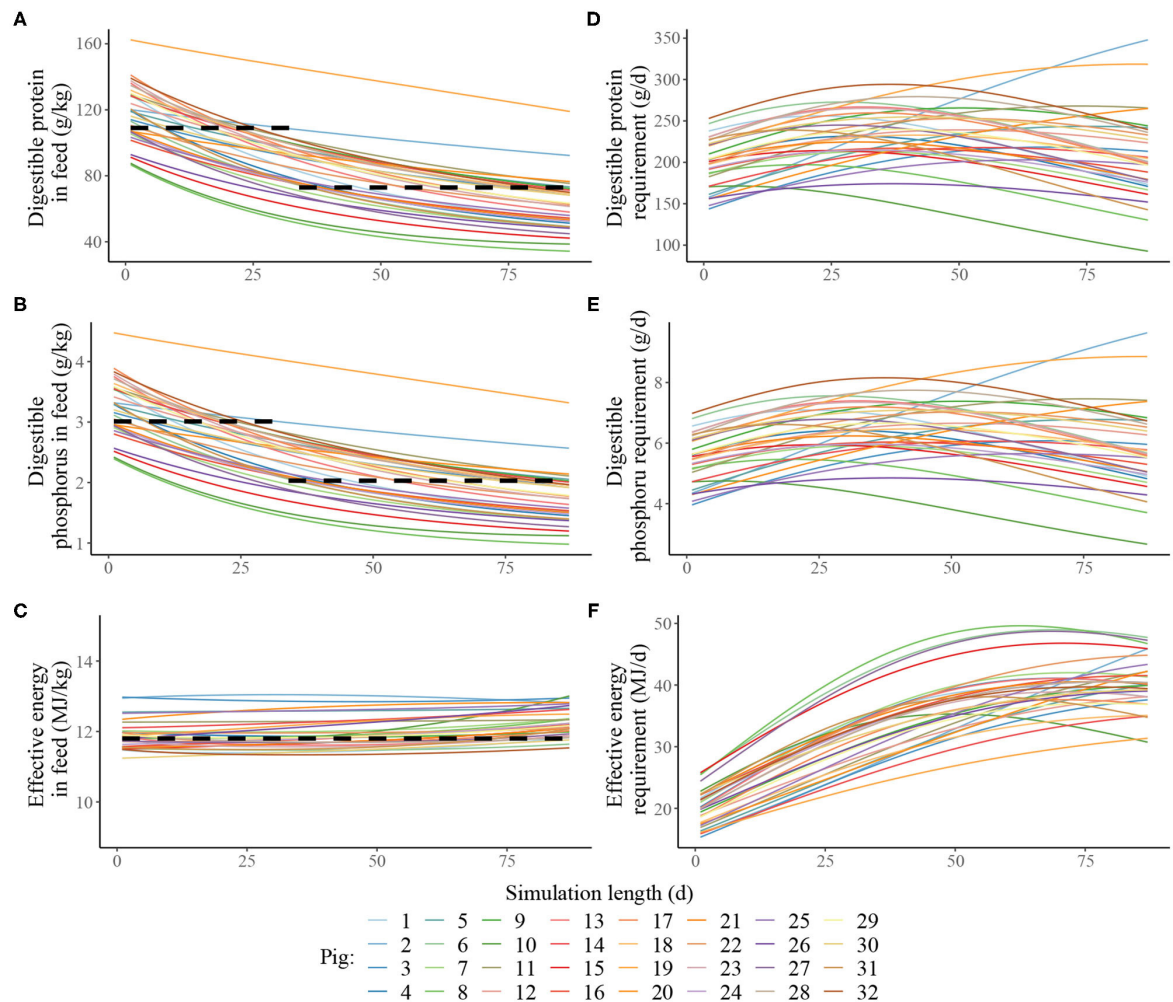
Estimated nutritional composition of the feeds in either two-phase feeding strategy (dashed black line, Scenario 3) or precision feeding strategy (solid lines; Scenario 4) given to a pig population in terms of: **(A)** digestible protein (g/kg); **(B)** digestible phosphorus (g/kg); **(C)** effective energy (MJ/kg); estimated daily nutrient requirement for maintenance and growth of each of the thirty-two pigs in the population in terms of: **(D)** digestible protein (g/d); **(E)** digestible phosphorus (g/d); **(F)** effective energy (MJ/d). For a detailed description of the simulated scenarios, see section Simulated Feeding Scenarios.

Estimated nutritional composition of the feeds in S3 and S4 (in terms of digestible N^*^, digestible P and effective energy), together with accompanying estimated daily nutrients requirements for maintenance and growth of each pig in the heterogenous population is given in Figure 3. In the context of the pig population, the precision feeding strategy (S4) also resulted in gradual decreases in digestible N^*^ and digestible P feed contents over time for each pig; the effective energy content also remained largely unchanged over time for each pig. There were notable differences in nutrient requirements of individual pigs, which were reflected in the differences in the estimated nutritional composition of the individualised feeds. For example, on the first day, the kg of feed offered to the pig with the lowest nutrient requirements was estimated to contain 145 g of crude N^*^, 86.7 of digestible N^*^, 4.78 g of total P, 2.39 g of digestible P and 11.2 MJ of effective energy, while the kg of feed offered to the pig with the highest nutrient requirements pig was estimated to contain 271 g of crude N^*^, 162 of digestible N^*^, 8.96 g of total P, 4.48 g of digestible P and 13.0 MJ of effective energy.

### Comparison of Growth Performance and Nutrient Excretion

A summary of the growth performance indicators calculated in the context of S1–S4 is given in Table 3. Relative to S1, S2 resulted in: 0.270% decrease in ADFI; 0.570% increase in ADG; 0.834% decrease in FCR; 1.19% increase in average daily N^*^ retention; 0.964% decrease in daily *L* retention; 0.902% increase in final N^*^ weight; and 0.949% decrease in final *L* weight. Relative to S3, S4 resulted in [mean (SD)]: 1.31 (3.38)% decrease in ADFI; 1.76 (3.32)% increase in ADG; 3.64 (7.04)% decrease in FCR; 3.12 (5.36)% increase in average daily N^*^ retention; 2.43 (4.54)% decrease in daily *L* retention; 2.13 (3.80)% increase in final N^*^ weight; and 2.19 (3.91)% decrease in final *L* weight.

**Table 3:** Summary statistics of average daily feed intake (ADFI); average daily gain (ADG); feed conversion ratio (FCR); protein (N^*^) retention; lipid (*L*) retention; final protein (N^*^) weight; and final lipid (*L*) weight in each of the four simulated scenarios in terms of mean values (S1 and S2 for the average pig) and mean (SD) values (S3 and S4 for the population of pigs).

	**Simulated Scenario**			
**Trait**	**S1**	**S2**	**S3**	**S4**
ADFI (kg/pig)	2.97	2.96	2.84 (0.359)	2.81 (0.403)
ADG (kg/pig)	1.03	1.04	0.982 (0.0949)	1.00 (0.0944)
FCR (kg/kg/pig)	2.88	2.86	2.89 (0.277)	2.82 (0.356)
N^*^ retention (kg/d/pig)	161	163	147 (18.6)	153 (21.4)
L retention (kg/d/pig)	325	322	323 (67.1)	317 (72.4)
Final N^*^ weight (kg/pig)	19.9	20.1	18.8 (2.02)	19.3 (2.24)
Final *L* weight (kg/pig)	28.6	28.4	31.5 (6.36)	31.0 (6.82)

*For a detailed description of the simulated scenarios, see section Simulated Feeding Scenarios*.

**Figure 4:**
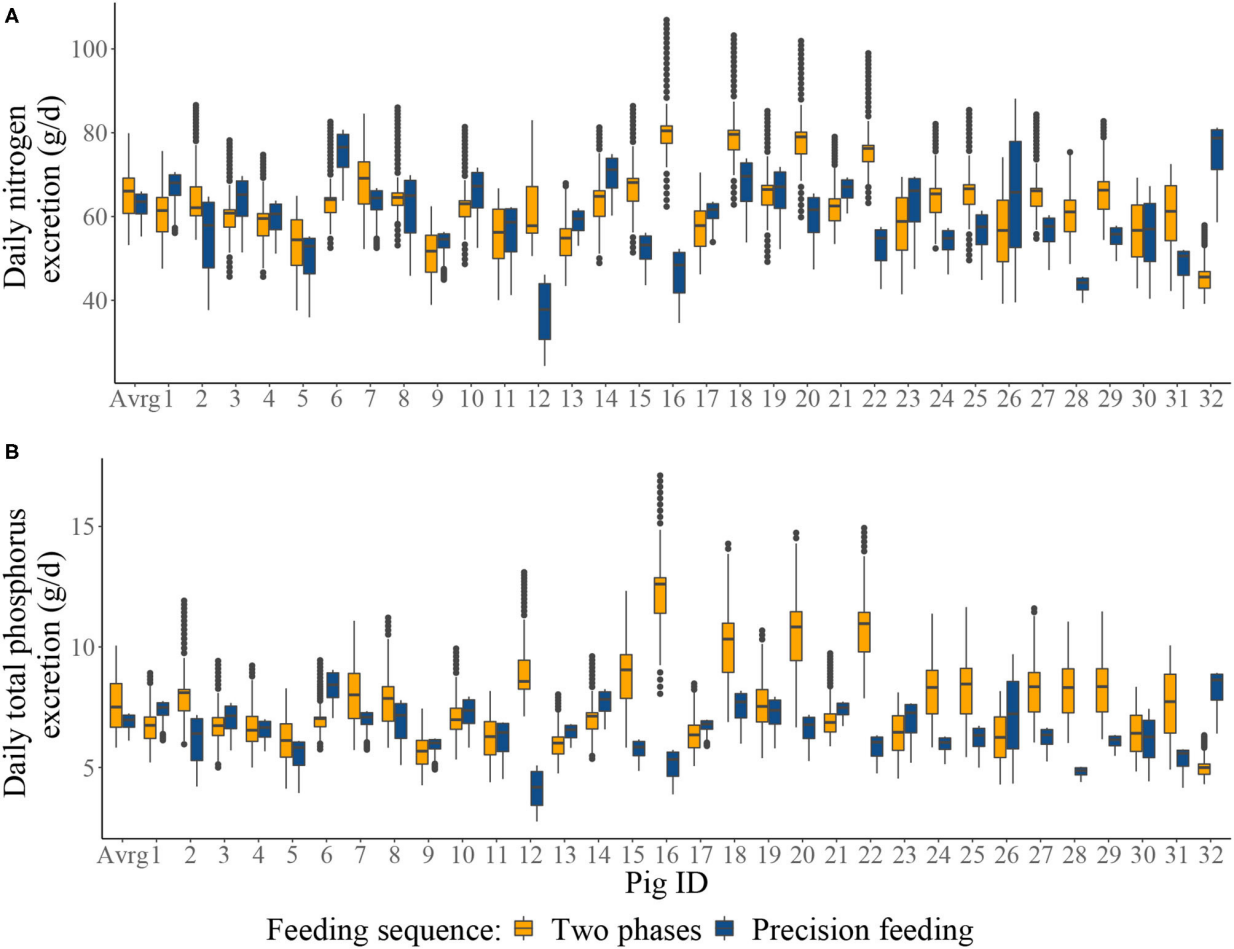
Boxplots of: **(A)** the individual daily nitrogen excretion (g/d); and **(B)** the individual daily total phosphorus excretion from two-phase feeding strategy or precision feeding strategy offered either to the average pig (Avrg) or each of the thirty-two pigs in the population (1–32). For a detailed description of the simulated scenarios, see section Simulated Feeding Scenarios.

Summary of the daily excretion of N and P over time for pigs considered in S1–S4 is given in Figure 4. Cumulative N and P balances calculated in the context of S1–S4 are given in Table 4. Relative to S1, S2 resulted in: 4.04% decrease in N intake; 0.858% increase in N retention; 8.25% decrease in N excretion; 3.93% decrease in total P intake; 1.04% increase in P retention; and 9.17% decrease in total P excretion. Relative to S3, S4 resulted in [mean (SD)]: 10.3 (23.9)% decrease in N intake; 2.98 (5.05)% increase in N retention; 22.5 (42.6)% decrease in N excretion; 10.0 (23.6)% decrease in total P intake; 4.35 (7.65)% increase in P retention; and 22.9 (40.2)% decrease in total P excretion.

**Table 4:** Calculated nitrogen (*N*) and phosphorus (P) balances in each of the four simulated scenarios in terms of mean values (S1 and S2 for the average pig) and mean (SD) values (S3 and S4 for the population of pigs).

	**Simulated Scenario**			
**Trait**	**S1**	**S2**	**S3**	**S4**
Cumulative N intake (kg/pig)	5.16	4.95	5.51 (0.710)	5.12 (0.751)
Cumulative N retention (kg/pig)	2.31	2.33	2.33 (0.273)	2.41 (0.307)
Cumulative N output (kg/pig)	2.85	2.62	3.18 (0.710)	2.71 (0.460)
Cumulative total P intake (kg/pig)	1.08	1.03	1.15 (0.148)	1.07 (0.154)
Cumulative P retention (kg/pig)	0.481	0.486	0.481 (0.0541)	0.506 (0.716)
Cumulative total P output (kg/pig)	0.599	0.544	0.669 (0.142)	0.564 (0.0828)

*For a detailed description of the simulated scenarios, see section Simulated Feeding Scenarios*.

## Discussion

### Estimation of the Unobserved Traits From Data

In practise, it is not possible to collect individual sequential measurements of the traits that determine growth potential and body composition, such as the growth of protein or lipid in growing-finishing pig systems (13–15). Yet, estimates of these traits are required to accurately estimate individual nutrient requirements (63). In this context, there is substantial research interest in developing mathematical models that utilise sequential data on individual bodyweight and feed consumption from electronic feeding and weighing stations to estimate these unobserved traits (4, 8, 64). To date, approaches to estimate the growth of protein have been developed, but the growth of the remaining main body chemical components (i.e., lipid, water, ash) has been largely overlooked, which could impact the estimation of the nutrient requirements needed to deliver tailored feeding strategies. In this chapter, an inferential approach utilising the concepts of inverse modelling (13, 65) was developed to estimate altogether the growth of the four main body chemical components (protein, water, lipid, and ash) from sequential bodyweight data that is typically available for precision feeding purposes. Joint estimation is preferred, as it ensures that all parameters that estimated traits are mutually consistent with the observed individual data (29). Accordingly, the estimates obtained via this approach could be used to formulate data-driven feeding strategies that more optimally match nutrient supply to the demand of pigs.

One of the main building blocks of the developed approach concerned a mathematical description of the relationship between protein weight and bodyweight, which consequently informs protein deposition. There is a considerable body of evidence suggesting that the relationship between these traits is approximately allometric (66–72). In light of this empirical evidence, the allometric model was chosen to describe the relationship between protein weight and bodyweight. This is in contrast with previous precision feeding studies, which suggest alternative ways of relating these traits, including isometric, quadratic and Gompertz relationships (5, 19). However, these models are inconsistent with the aforementioned empirical evidence and thus, were not considered further in this chapter. The remaining body elements were related to protein, based on similar well-established allometric scaling rules supported by the view that lipid-free dry matter is considered to be one of the best indicators of the growth progress (29, 34–36). While these rules seem plausible for pigs kept in high-standard livestock production systems, modifications to the allometric body composition models could be needed if there is evidence suggesting that the data originated from pigs faced with severe limitations in the availability of nutrient resources.

### Characterisation of the Distributions and Correlations of Unobserved Traits

In the context of pig production data, population and individual level trait estimation is typically carried out within a framework based on hierarchical regression models (19, 73). Under this framework, the overall quality of inferences could be negatively impacted by having to directly estimate multiple variance-covariance parameters (27), which can be challenging due to data limitations (9). The technical difficulties associated with this estimation procedure are the main reason why several studies make various working assumptions that neglect trait correlations (20, 54, 61). However, as highlighted by Pomar et al. (22), this is undesirable as it could lead to an overestimation of the trait variation in a population. In an attempt to alleviate these concerns, the developed approach to estimate population and individual level traits described in this paper, shifted away from hierarchical regression modelling in favour of an alternative framework based on separately inferring individual level trait distributions, which were then scaled up to obtain population level traits. This alternative framework does not necessitate an explicit specification of the aforementioned variance-covariance parameters (27). Thus, reducing the number of assumptions and the number of parameters that need to be estimated should increase the ability to adequately characterise the traits of individual pigs, which should lead to a greater understanding of the impact of such differences on the estimation of population averages (74).

Overall, the developed approach to characterise unobserved traits from bodyweight data on growing (Large White × Landrace) × Pietrain barrows and gilts converged to biologically plausible estimates for most pigs in the population (65, 75). There was considerable variation in the estimated traits among pigs, but very few individuals were identified as potential outliers. However, it is difficult to ascertain if these potential outliers are a result of genetics, environment, feeding and management practises, a combination of some of these factors (76), or data limitations (27). Moreover, it is also important to note that since the parameters on body composition were estimated conditional on the bodyweight parameter estimates and without any additional data, some of these estimates could carry increased uncertainty and have limited biological interpretability (29).

As highlighted by Gauthier et al. (77), mathematical models applied in the context of precision feeding should be able to process both more extensive “historical” data, covering longer timescales and less extensive “real-time” data, covering shorter timescales. When dealing with the latter type of data, it is likely that there will be additional uncertainty in the estimates of body composition and thus, in the estimates of nutrient requirements. The purpose of the current study was to hindcast the nutrient excretion of growing-finishing pigs under differing hypothesised feeding strategies to quantify the differences in the nutrient excretion between these different feeding strategies. Consequently, the developed approach was not tested in the context of “real-time” data, but such evaluations could be an area of future research.

### Mechanism of Feed Intake Regulation and Consequences on Nutrient Intake

Recent advances in engineering enable the delivery of feeds, that can be tailored to the needs of individual pigs at a particular point in time (78, 79). It is expected that there would be improvement in feed and nutrient utilisation efficiency if such individualised, data-driven feeding strategies are implemented. In this study, simulation modelling was utilised to illustrate how the estimated variation in individual growth potential and body composition traits could be incorporated in a proposed precision feeding strategy. Specifically, simulations were carried out to assess growth performance and nutrient excretion in the context of a precision feeding plan with daily adjustments and a commercial two-phase feeding plan that did not adapt to real-time animal performance. However, before describing the outcomes of these simulations, it is important to highlight some of the key assumptions concerning how growth was simulated, as these assumptions predetermine the consequent assessments.

In the simulations, the actual growth of pigs was allowed to differ from the estimated potential growth. These differences were largely conditional upon feed composition. Specifically, it was assumed that when the feeds were deficient in either energy or protein, the pigs would attempt to increase their feed consumption according with the previous empirical evidence (59, 80–83). For the purposes of this study, no constraint was assumed to prevent the pigs from meeting their requirements for these two nutrient resources. In reality, however, it is likely that some constraints would operate and prevent the animals from achieving these goals (84, 85). This potential compensatory feed consumption was assumed to be absent in cases when P was the most deficient nutrient to reflect the current knowledge of feed intake regulation in the context of this nutrient (49, 60). In those cases, it was assumed that feed intake could be predicted solely from the estimated energy requirements and energy content of feeds. If the nutritional deficiency triggers attempts to eat for the most deficient nutrient resource, then a possible consequence of this feed intake mechanism would be the excess consumption of the remaining nutrients, leading to their excess excretion (62). In scenarios when feeds were no longer deficient, there was no attempt to correct for any potential imbalances in the body composition as a result of uncertainty surrounding the phenomenon of compensatory growth, especially in relation to the correction of the lipid to protein ratio in the body (86–88).

### Comparison of Feeding Strategies Regarding Growth Performance and Nutrient Excretion

The aforementioned simulations were structured to assess the potential advantages (or disadvantages) of precision feeding strategies as measured by the average of individual responses in a population and by the response of an assumed average pig in a population, as it is appreciated that there could be notable differences between these two responses (20). These differences are conditional upon the levels of heterogeneity in the population (24). Note that the present simulations serve mainly as an illustration of the developed approach to estimate individual level variation in unobserved traits and assess deviations from the population average. Additional simulations could be carried out as sensitivity analysis or to evaluate different feeding scenarios.

In scenarios simulating the average pig in the population, which would only represent a population if it were homogenous, the precision feeding strategy led to an approximate ten percent decrease in N and P excretion compared to the typical two-phase feeding strategy. In this case, the higher nutrient excretion from the typical two-phase feeding strategy could be attributed to periods of over-supplementation. Extending the comparisons to the heterogeneous pig population demonstrated an even greater decrease in N and P excretion in the precision feeding strategy compared to the two-phase feeding strategy that targeted nutrient requirements of the average animal (~20% reduction). These estimates are consistent with previous studies evaluating precision feeding strategies, which reported an average reduction in N and P excretion ranging from approximately ten to forty percent (10, 12, 89), although those studies focussed on evaluating individualised feeding strategies against three-phase feeding sequences. The additional decrease in nutrient excretion observed in the context of the heterogenous pig population could be explained by what happens to the pigs whose nutrient requirements differed from those of the average pig. When offered the phase feeding strategy, the pigs with lower nutrient requirements were oversupplied, leading to notable periods of excess excretion that was mitigated by the precision feeding strategy. The converse was also true implying that the pigs with higher nutrient requirements were excreting more nutrients when offered the precision feeding strategy due to the inefficiencies associated with higher nutrient intakes. Both feeding strategies resulted in comparable growth performance, which is consistent with the previous literature (10, 12, 89). However, there were some differences between growth performance in the context of the two feeding strategies under consideration. Specifically, the precision feeding strategy led to small increases in average daily gain and protein retention, and small decreases in average daily feed intake, feed conversion ratio and lipid retention when compared to the typical two-phase feeding strategy. Again, these differences were magnified in the context of the heterogenous pig population due to the individual level variation in nutrient requirements, which led to more severe periods of both under-supplementation and over-supplementation for some animals.

Note that in the precision feeding strategies under consideration, two feed components (protein and phosphorus) were subject to considerable adjustments over time. It was assumed that the current practise of blending high-nutrient and low-nutrient basal feeds would be largely compatible with such adjustments, although this is not fully guaranteed. To ensure universality, blending three basal feeds is likely to be needed (90).

## Conclusions

Alternative data-driven approaches to estimate individual level variation in unobserved traits using the available data on *BW* were developed. The key advantages of these alternative approaches relate to the improvements made in terms of characterisation of the traits of individual pigs, which should also lead to a greater understanding of the impact of such differences on the estimation of population averages. This was achieved through: (1) a more comprehensive description of the growth potential and body composition; and (2) a reduction in the number of parameters needed to be estimated compared to the typical hierarchical regression models. Consequently, these alternatives approaches were incorporated in a proposed precision feeding modelling framework to quantify the differences in the nutrient excretion between individualised feeding strategies and standard feeding strategies. It was found that the implementation of individualised feeding strategies could notably reduce nutrient excretion in pig populations, which supports the earlier findings by other researchers. The main outstanding challenge relates to whether the developed approaches are applicable in the context of ‘real-time' data on bodyweight of pigs that has been collected over shorter periods of time, than those examined in this study which covered the entire growing-finishing phase of growth. Overall, the outcomes of this study should increase the ability to accurately match nutrient supply to the demand of animals by building a more comprehensive picture of their individual nutrient requirements. Moreover, the proposed methodology could also be relevant in the context of selective breeding focussing on improving feed efficiency, such as in the case of residual feed intake-based genetic selection.

## Data Availability Statement

The data analysed in this study was obtained from the National Research Institute for Agriculture, Food and Environment (INRAE) Pig Physiology and Phenotyping Experimental Facility (UE3P) in Saint-Gilles (France) (https://doi.org/10.15454/1.5573932732039927E12). Requests to access these datasets should be directed to Dr Ludovic Brossard, ludic.brossard@inrae.fr.

## Ethics Statement

Ethical review and approval was not required for the animal study because the empirical data used in this paper were not generated in this study. The data originated from animals treated under normal husbandry procedures and for this reason no Institutional or other relevant ethics board approval was required for its collection.

## Author Contributions

MM led the development and implementation of the approaches in R and drafted the first version of the manuscript. JF conceptualised the algorithms for the estimation of unobserved traits. IK managed both the BBSRC and Feed-a-Gene projects which supported financially the paper development and MM studies. This paper is a part of MM doctoral thesis. All authors contributed equally to the inception of the study, its development, interpretation and conclusions, and contributed equally to the development and finalisation of the manuscript.

## Conflict of Interest

The authors declare that the research was conducted in the absence of any commercial or financial relationships that could be construed as a potential conflict of interest.

## Publisher's Note

All claims expressed in this article are solely those of the authors and do not necessarily represent those of their affiliated organizations, or those of the publisher, the editors and the reviewers. Any product that may be evaluated in this article, or claim that may be made by its manufacturer, is not guaranteed or endorsed by the publisher.
