# Supplementary material for: A Novel Estimation of Unobserved Pig Growth Traits for the Purposes of Precision Feeding Methods
Source: Front Vet Sci. 2021 Jul 29;8:689206. doi: 10.3389/fvets.2021.689206 (PMC8360350; doi:10.3389/fvets.2021.689206)
Supplement: Supplementary file 1 [file Table_1.DOCX]

**Supplementary Material A:** Prior distributions of the main unknown parameters in the model for growth potential and body composition

$$BW_{t}=BW_{m}\times\exp\left( -\ln\left( \frac{BW_{m}}{BW_{in}} \right)\times\exp\left( -\frac{t-t_{0}}{B} \right) \right) (kg)$$

$$N_{t}^{*}\boldsymbol{=}N_{m}^{*}\times\left( \frac{BW_{t}}{BW_{m}} \right)^{\frac{\log\left( {N_{m}^{*}}/{N_{in}^{*}} \right)}{\log\left( {BW_{m}}/{BW_{in}} \right)}} (\mathrm{kg})$$

$$L_{t}= L_{m}\times\left( \frac{BW_{t}}{BW_{m}} \right)^{\frac{\log\left( {L_{m}}/{L_{in}} \right)}{\log\left( {BW_{m}}/{BW_{in}} \right)}} (\mathrm{kg})$$

| **Parameter** | **Prior distribution** | **Justification** |
| --- | --- | --- |
| ${BW}_{m}$ | ${BW}_{m} \sim U(100,600)$ | The hypothetical body size at maturity could be 3-4 times the typical size of an animal at slaughter (90-120k kg) (Strathe et al., 2010); since there is scarce data on body size at maturity (Whittemore, 1994, Filipe et al., 2018), it was assumed that all values within the listed range are equally likely |
| $B$ | $B \sim U(10, 1000)$ | If kept for sufficiently long enough, the weight curve of a pig has been reported to flatten at around 18 months from birth (i.e., 548 d) in older breeds (Reiland, 1978); thus the range from 10 to 1000 should be sufficiently broad enough to capture controlling how fast the weight at maturity is reached |
| $N_{m}^{*}$ | $N_{m}^{*} \sim U\left( 0.08\times BW_{m}, 0.25\times BW_{m} \right)$ | A flat, vaguely informative prior (Gelman et al., 2017) derived from extrapolating from the expected body composition at the slaughter weight (Kyriazakis and Whittemore, 2006, de Lange et al., 2003) |
| $N_{in}^{*}$ | $N_{in}^{*} \sim U(0.12\times0.95\times BW_{in},0.20\times0.95\times BW_{in})$ | A flat, vaguely informative prior (Gelman et al., 2017) based on the expected body composition of pigs at the start of the growing phase (Kyriazakis and Whittemore, 2006, de Lange et al., 2003) |
| $L_{m}$ | $L_{m}=\pi\times N_{m}^{*};\pi=\left( \frac{BW_{m}}{N_{m}^{*}}-4.23 \right)$ | Due to the assumed allometric relationships (Emmans and Kyriazakis, 1995, Emmans, 1997, Emmans and Kyriazakis, 1997, Filipe et al., 2018), it is possible to directly calculate $L_{m}$ from the remaining parameters |
| $L_{in}$ | $L_{in} \sim U(0.05\times0.95\times BW_{in}, 0.25\times0.95\times BW_{in})$ | A flat, vaguely informative prior (Gelman et al., 2017) based on the expected body composition of pigs at the start of the growing phase (Kyriazakis and Whittemore, 2006, de Lange et al., 2003) |

**References**

DE LANGE, C. F. M., MOREL, P. C. H. & BIRKETT, S. H. 2003. Modeling chemical and physical body composition of the growing pig. *J Anim Sci,* 81**,** E159-E165. DOI: <https://doi.org/10.2527/2003.8114_suppl_2E159x>

EMMANS, G. C. 1997. A method to predict the food intake of domestic animals from birth to maturity as a function of time. *J Theor Biol,* 186**,** 189-199. DOI: <https://doi.org/10.1006/jtbi.1996.0357>

EMMANS, G. C. & KYRIAZAKIS, I. 1995. A general method for predicting the weight of water in the empty bodies of pigs. *Animal Sci,* 61**,** 103-108. DOI: <https://doi.org/10.1017/S1357729800013576>

EMMANS, G. C. & KYRIAZAKIS, I. 1997. Models of pig growth: problems and proposed solutions. *Livest Prod Sci,* 51**,** 119-129. DOI: <https://doi.org/10.1016/S0301-6226(97)00061-4>

FILIPE, J. A. N., LEINONEN, I. & KYRIAZAKIS, I. 2018. The quantitative principles of animal growth. *In:* MOUGHAN, P. J. & HENDRIKS, W. H. (eds.) *Feed Evaluation Science.* 1 ed. Wageningen: Wageningen Academic Publishers.

GELMAN, A., SIMPSON, D. & BETANCOURT, M. 2017. The prior can often only be understood in the context of the likelihood. *Entropy,* 19**,** 555. DOI: <https://doi.org/10.3390/e19100555>

KYRIAZAKIS, I. & WHITTEMORE, C. T. 2006. *Whittemore's Science and Practice of Pig Production,* Oxford, UK, Blackwell Publishing

REILAND, S. 1978. Growth and skeletal development of the pig. *Acta Radiol Suppl,* 358**,** 15-22. <http://europepmc.org/abstract/MED/233594>

STRATHE, A. B., DANFÆR, A., SØRENSEN, H. & KEBREAB, E. 2010. A multilevel nonlinear mixed-effects approach to model growth in pigs. *J Anim Sci,* 88**,** 638-649. DOI: <https://doi.org/10.2527/jas.2009-1822>

WHITTEMORE, C. T. 1994. Causes and consequences of change in the mature size of the domestic pig. *Outlook Agr,* 23**,** 55-59. DOI: <https://doi.org/10.1177/003072709402300110>

**Supplementary Material 4B:** Comparison of growth performance and nutrient excretion with no potential outlier pigs

| **Trait** | **S1** | **S2** | **S3** | **S4** |
| --- | --- | --- | --- | --- |
| ADFI (kg/pig) | 2.97 | 2.96 | 2.88 (0.337) | 2.86 (0.378) |
| ADG (kg/pig) | 1.03 | 1.04 | 0.984 (0.0896) | 0.994 (0.0911) |
| FCR (kg/kg/pig) | 2.88 | 2.86 | 2.93 (0.257) | 2.88 (0.296) |
| $N^{*}$ retention (kg/d/pig) | 161 | 163 | 148 (19.0) | 151 (21.2) |
| $L$ retention (kg/d/pig) | 325 | 322 | 329 (65.9) | 325 (69.0) |
| Final $N^{*}$ weight (kg/pig) | 19.9 | 20.1 | 18.9 (2.11) | 19.2 (2.29) |
| Final $L$ weight (kg/pig) | 28.6 | 28.4 | 31.9 (6.28) | 31.6 (6.56) |

**Table 1.** Summary statistics of average daily feed intake (ADFI); average daily gain (ADG); feed conversion ratio (FCR); protein ($N^{*}$) retention; lipid ($L$) retention; final protein ($N^{*}$) weight; and final lipid ($L$) weight in each of the four simulated scenarios in terms of mean values (S1 and S2) and mean (SD) values (S3 and S4). For a detailed description of the simulated scenarios, see section Simulated feeding scenarios

| **Trait** | **S1** | **S2** | **S3** | **S4** |
| --- | --- | --- | --- | --- |
| Cumulative N intake (kg/pig) | 5.16 | 4.95 | 5.58 (0.667) | 5.06 (0.731) |
| Cumulative N retention (kg/pig) | 2.31 | 2.33 | 2.32 (0.279) | 2.38 (0.311) |
| Cumulative N output (kg/pig) | 2.85 | 2.62 | 3.26 (0.692) | 2.68 (0.423) |
| Cumulative total P intake (kg/pig) | 1.08 | 1.03 | 1.17 (0.139) | 1.06 (0.150) |
| Cumulative P retention (kg/pig) | 0.481 | 0.486 | 0.485 (0.0625) | 0.502 (0.0858) |
| Cumulative total P output (kg/pig) | 0.599 | 0.544 | 0.685 (0.138) | 0.558 (0.0809) |

**Table 2.** Calculated nitrogen (N) and phosphorus (P) balances in each of the four simulated scenarios in terms of mean values (S1 and S2) and mean (SD) values (S3 and S4). For a detailed description of the simulated scenarios, see section Simulated feeding scenarios.
